# Long non-coding RNA ACTA2-AS1 suppresses metastasis of papillary thyroid cancer via regulation of miR-4428/KLF9 axis

**DOI:** 10.1186/s13148-023-01622-6

**Published:** 2024-01-09

**Authors:** Shuhui Wu, Jingjing Zhu, Tingting Jiang, Ting Cui, Qi Zuo, Guibin Zheng, Guojun Li, Jieyu Zhou, Xiang Chen

**Affiliations:** 1 Department of Otorhinolaryngology, Shanghai Baoshan Hospital of Integrated Traditional Chinese and Western Medicine, Baoshan Hospital Affiliated to Shanghai University of Traditional Chinese Medicine, Shanghai, People’s Republic of China; 2 Department of Thyroid Surgery, Shanghai Baoshan Hospital of Integrated Traditional Chinese and Western Medicine, Baoshan Hospital Affiliated to Shanghai University of Traditional Chinese Medicine, Shanghai, People’s Republic of China; 3 Department of Head and Neck Surgery, The University of Texas MD Anderson Cancer Center, Houston, TX 77030 USA; 4 Department of Thyroid Surgery, The Affiliated Yantai Yuhuangding Hospital of Qingdao University, Yantai, 264000 Shandong China; 5 Department of Otolaryngology-Head and Neck Surgery, Shanghai Ninth People’s Hospital, Shanghai Jiaotong University School of Medicine, Shanghai, China; 6 Ear Institute, Shanghai Jiaotong University School of Medicine, Shanghai, China; 7 Shanghai Key Laboratory of Translational Medicine on Ear and Nose Diseases, Shanghai, China

**Keywords:** lncRNA, ACTA2-AS1, miR-4428, KLF9, Papillary thyroid carcinoma

## Abstract

**Background:**

Metastasis is the primary cause of recurrence and death in patients with papillary thyroid carcinoma (PTC). LncRNA ACTA2-AS1, a long non-coding RNA, acts as a tumor suppressor in multiple types of human malignancies, while the role of ACTA2-AS1 in PTC metastasis remains unclear.

**Methods:**

The ACTA2-AS1 expression in PTC tissues was analyzed. The sponged roles of ACTA2-AS1 via miR-4428/KLF9 axis were identified using starBase tool. The function of ACTA2-AS1 in PTC was performed with in vitro and in vivo experiments. The correlation between DNA methylation and mRNA expressions of these gene in the TCGA dataset was explored.

**Results:**

ACTA2-AS1 expression was downregulated in PTC tissues without metastasis and further decreased in PTC tissues with lymph node metastasis compared with that in normal tissues. Functionally, the overexpression of ACTA2-AS1 inhibited the growth, proliferation, and invasion of PTC cells, whereas its depletion exerted opposite effect. In vivo, ACTA2-AS1 expression inhibited PTC metastasis. Furthermore, ACTA2-AS1 acted as a competing endogenous RNA for miR-4428, thereby positively regulating the expression of miR-4428 target gene, *KLF9*. Finally, miR-4428 overexpression enhanced invasive potential of PTC cells and significantly weakened the effects of ACTA2-AS1 on promotion and inhibition of KLF9 expression as well as invasive ability of PTC cells, respectively. In the TCGA dataset, the methylation level of ACTA2-AS1 was significantly correlated with its mRNA expression (*r* = 0.21, *p* = 2.1 × e^−6^).

**Conclusions:**

Our findings demonstrate that ACTA2-AS1 functions as a tumor suppressor in PTC progression at least partly by regulating the miR-4428-dependent expression of *KLF9*.

**Graphical abstract:**

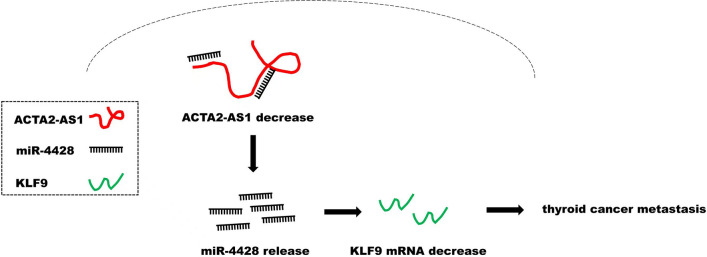

**Supplementary Information:**

The online version contains supplementary material available at 10.1186/s13148-023-01622-6.

## Background

Thyroid carcinoma (TC) is the most frequently diagnosed malignancy of the endocrine system, with an estimated 586,000 new cases and 43,600 deaths worldwide in 2020 [1]. The incidence of TC has increased continuously over the past few decades [2, 3] while TC-associated mortality is approximately 3 and 5 per million among men and women, respectively [1]. TC originates from follicular cells (approximately 90% of cases) or parafollicular cells (approximately 10% of cases) [3]. TCs of follicular origin comprise four types, among which papillary TC (PTC) is the most frequent histologic subtype, accounting for 80–90% of the total [4]. Several risk factors for PTC have been identified to date, including ionizing radiation, abnormal iodine intake, and genetic factors [5, 6]. However, the molecular mechanisms underlying PTC development remain unclear.

Long non-coding RNAs (lncRNAs) are initially thought to be merely by-products of transcription; however, they are now known to be functional RNA transcripts > 200 nucleotides in length which play critical roles in the etiology of a variety of human diseases [7, 8]. LncRNAs, such as HOTAIR, PCGEM1, H19, and UCA1, are dysregulated in many types of tumor tissues and can function either as oncogenes or tumor suppressors through regulating cancer cell growth, apoptosis, and invasion [9–11]. For instance, HOTAIR levels are increased in PTC tissues, which accelerates PTC progression through the upregulation of cyclin D2 expression [12, 13]. When upregulated, H19 facilitates epithelial–mesenchymal transition and PTC cell invasion via repressing the expression of E-cadherin, thus increasing expression of vimentin [14, 15]. The levels of ACTA2-AS1 are significantly decreased in human colon lung tissues. Meanwhile, its overexpression represses the proliferative and colony-forming abilities of cancer cells by decreasing BIM expression [16]. ACTA2-AS1 is also associated with tumor progression in lung and liver cancer [17–19]. However, the role of ACTA2-AS1 in PTC metastasis remains unclear.

MicroRNAs (miRNAs) comprise another class of functional ncRNAs with lengths ranging from 19 to 25 nucleotides. MiRNAs exert crucial regulatory effects in different types of human diseases. For instance, miR-4428 and miR-155 are dysregulated in many types of tumor tissues, while abnormal miRNA expression is correlated with tumorigenesis and poor prognosis [20, 21]. Additionally, miR-200a overexpression accelerates bladder cancer cell proliferation and invasion by targeting Dicer [22].

Interestingly, lncRNAs frequently exert their promotive effects on cancer progression by functioning as competing endogenous RNAs (ceRNAs), sponging miRNAs through miRNA-response elements (MREs) [23]. In this study, we found that ACTA2-AS1 levels were downregulated in metastatic PTC tissues, and that ACTA2-AS1 overexpression inhibited PTC metastasis through sponging miR-4428 and, consequently, upregulating KLF9 expression.

## Results

### Downregulation of ACTA2-AS1 expression in PTC

**Fig. 1:**
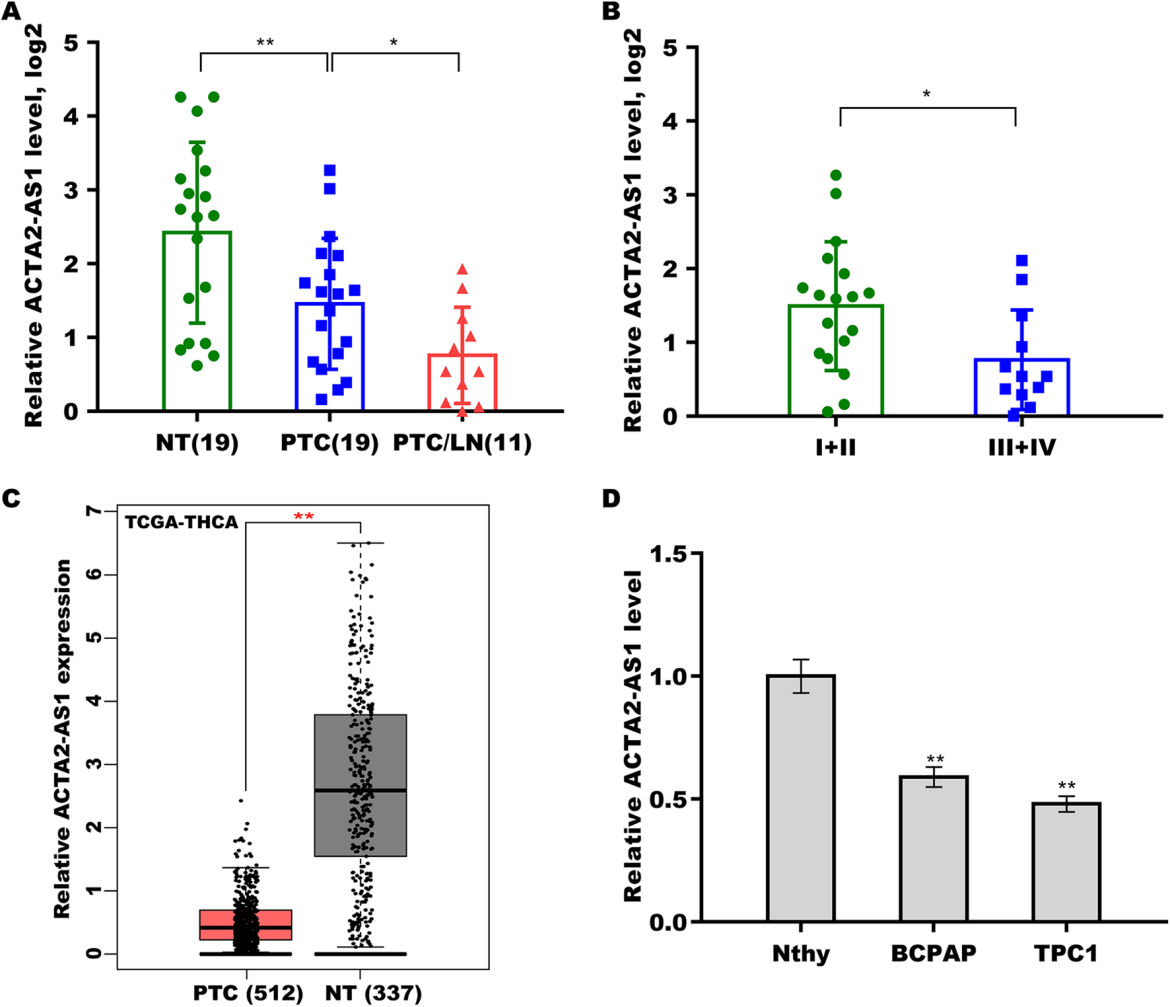
ACTA2-AS1 levels were downregulated in papillary thyroid carcinoma (PTC). **A** qRT-PCR analysis of the levels of ACTA2-AS1 in normal thyroid tissues (*n* = 19), PTC tissues without metastasis (*n* = 19), and PTC tissues with lymph node metastasis (*n* = 11). The data were log2-transformed (2^–ΔΔCt^). **B** qRT-PCR analysis of the levels of ACTA2-AS1 in stage I/II (*n* = 18) and stage III/IV (*n* = 12) PTC tissues. The data were log2-transformed (2^–ΔΔCt^). **C** The levels of ACTA2-AS1 were analyzed in 512 thyroid carcinoma tissues and 337 normal tissues in The Cancer Genome Atlas (TCGA)/Genotype-Tissue Expression (GTEx) datasets from GEPIA. **D** qRT-PCR analysis of ACTA2-AS1 levels in BCPAP, TPC1, and Nthy-ori 3–1 cells. *p < 0.05, **p < 0.01

Recent studies have demonstrated that ACTA2-AS1 inhibits tumor progression and mitigates resistance to chemotherapy in various cancers [16–19]. To address whether ACTA2-AS1 influences PTC metastasis, we assessed the levels of ACTA2-AS1 in 19 PTC tissues and matched normal tissues. As shown in Fig. 1A, compared with control tissues, ACTA2-AS1 level was significantly downregulated in PTC tissues. Moreover, the ACTA2-AS1 level was continuously downregulated in PTC tissues with lymph node metastasis (Fig. 1A). The ACTA2-AS1 level was lower in PTC tissues at clinical stages III/IV than in those at clinical stages I/II (Fig. 1B), indicating that ACTA2-AS1 expression is negatively correlated with PTC progression.

The ACTA2-AS1 expression was then analyzed based on data for 512 thyroid carcinoma tissues and 337 normal tissues in The Cancer Genome Atlas (TCGA)/Genotype-Tissue Expression (GTEx) datasets from the GEPIA webserver (http://gepia.cancer-pku.cn/index.html). As shown in Fig. 1C, ACTA2-AS1 expression was significantly decreased in PTC compared with that in normal tissues. ACTA2-AS1 expression was also lower in the PTC cell lines than in Nthy cells (Fig. 1D).

### Inhibition of ACTA2-AS1in the growth and proliferation of PTC cells

**Fig. 2:**
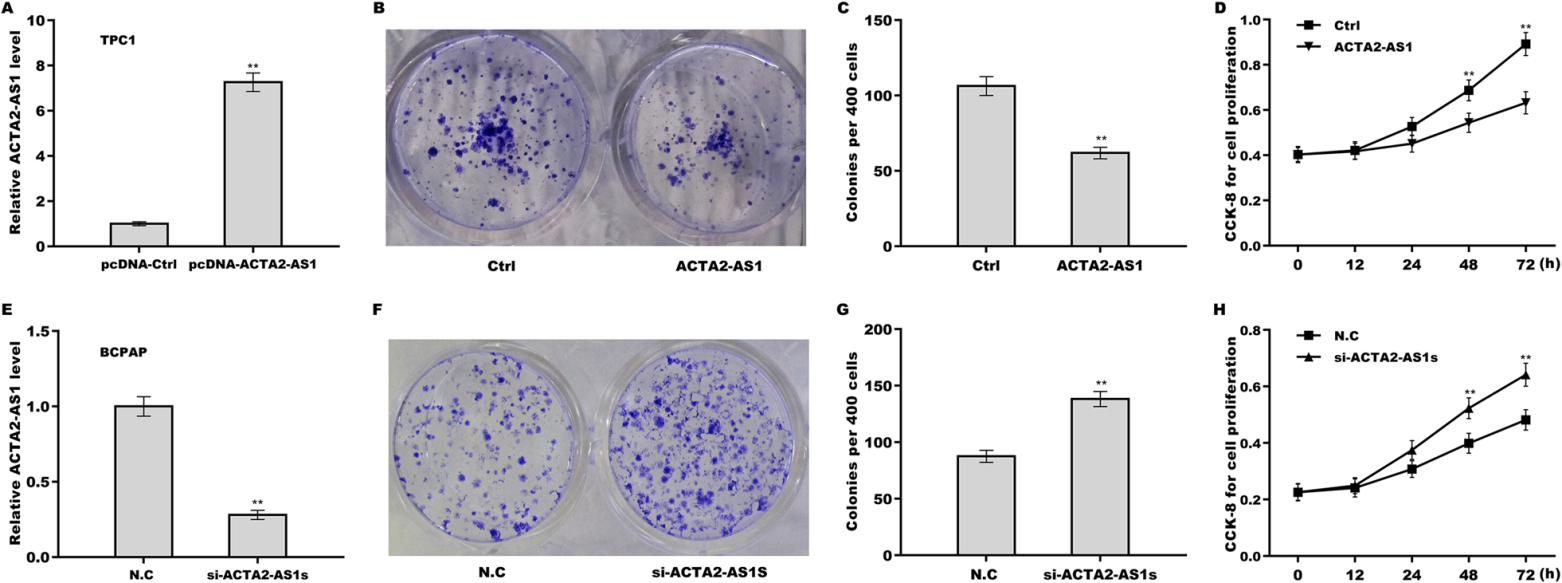
ACTA2-AS1 inhibited the growth and proliferation of papillary thyroid carcinoma (PTC) cells. **A** qRT-PCR analysis of ACTA2-AS1 levels in TPC1 cells overexpressing ACTA2-AS1. **B**, **C** The growth of ACTA2-AS1-overexpressing TPC1 cells was assessed using a colony formation assay. **D** The proliferative ability of ACTA2-AS1-overexpressing TPC1 cells was assessed by Cell Counting Kit-8 (CCK-8) assay. **E** qRT-PCR analysis of ACTA2-AS1 levels in ACTA2-AS1-knockdown BCPAP cells. **F**, **G** The growth of ACTA2-AS1-silenced BCPAP cells was assessed using a colony formation assay. **H** The proliferative ability of ACTA2-AS1-silenced BCPAP cells was assessed by CCK-8 assay. *p < 0.05, **p < 0.01

To assess the effect of ACTA2-AS1 on the growth and proliferation of PTC cells, ACTA2-AS1 was overexpressed in TPC1 cells (Fig. 2A) and silenced in BCPAP cells (Fig. 2E). The results of the colony formation assay revealed that upregulation of ACTA2-AS1 significantly repressed the growth of TPC1 cells (Fig. 2B, C), whereas its silencing elicited the opposite effect in BCPAP cells (Fig. 2F, G). Meanwhile, the results of the CCK-8 assay showed that ACTA2-AS1 overexpression suppressed TPC1 cell proliferation (Fig. 2D), whereas ACTA2-AS1 silencing enhanced the proliferative potential of BCPAP cells (Fig. 2H).

### Inhibition of ACTA2-AS1in PTC metastasis

**Fig. 3:**
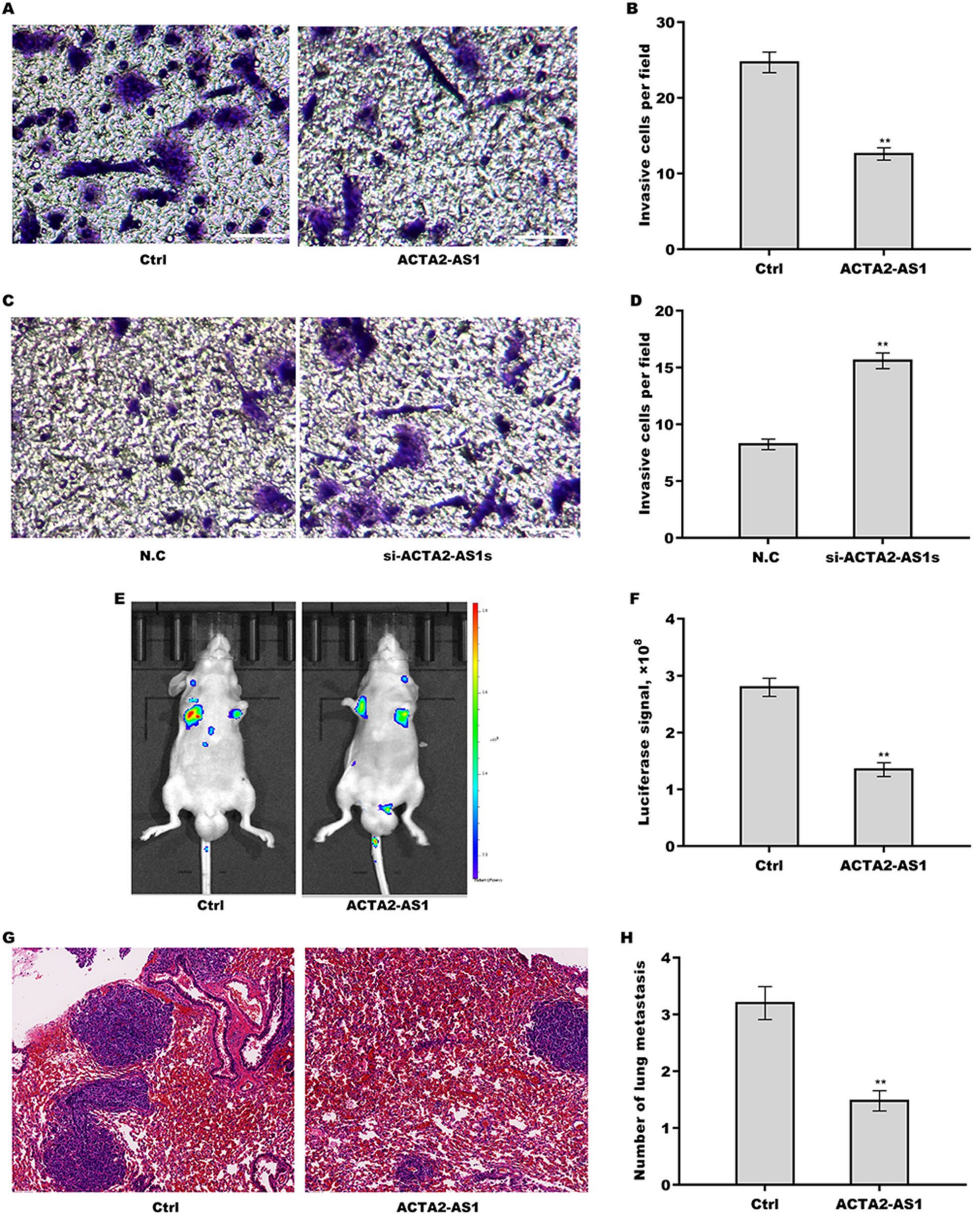
ACTA2-AS1 inhibited papillary thyroid carcinoma (PTC) metastasis. **A**, **B** Transwell invasion assay for the invasive ability of ACTA2-AS1-overexpressing TPC1 cells (**A**) and the respective quantitative analysis (**B**). Scale bar, 50 µm and magnification, 200× . **C**, **D** Transwell invasion assay for the invasive ability of ACTA2-AS1-silenced BCPAP cells (**C**) and the respective quantitative analysis (**D**). Scale bar, 50 µm and magnification, 200× . **E**, **F** ACTA2-AS1-overexpressing TPC1 cells were injected into nude mice via the tail vein (*n* = 3) and the extent of tumor metastasis was determined after 9 weeks using in vivo bioluminescent imaging. **G** Hematoxylin and eosin (H&E) staining was carried out to assess cancer metastasis to lung tissues. Scale bar, 100 µm and magnification, 100× . **H** Quantification of lung metastatic nodules. *p < 0.05, **p < 0.01

We explored the effect of ACTA2-AS1 on the invasive and metastatic potential of PTC cells. Transwell invasion assay revealed that ACTA2-AS1 overexpression significantly inhibited the invasive potential of TPC1 cells (Fig. 3A and B), whereas the silencing of ACTA2-AS1 greatly enhanced the invasive ability of BCPAP cells (Fig. 3C and D). To investigate the effect of ACTA2-AS1 on PTC metastasis, a lung metastasis model was established in mice through the injection of ACTA2-AS1-overexpressing TPC1 cells. As shown in Fig. 3E and F, ACTA2-AS1 overexpression significantly suppressed the lung metastasis of TPC1 cells. Moreover, the H&E staining indicated that ACTA2-AS1-overexpressing TPC1 cells formed fewer and smaller metastatic lung nodules when compared with untransfected TPC1 cells (Fig. 3G, H).

### ACTA2-AS1 function as a sponge of miR-4428

**Fig. 4:**
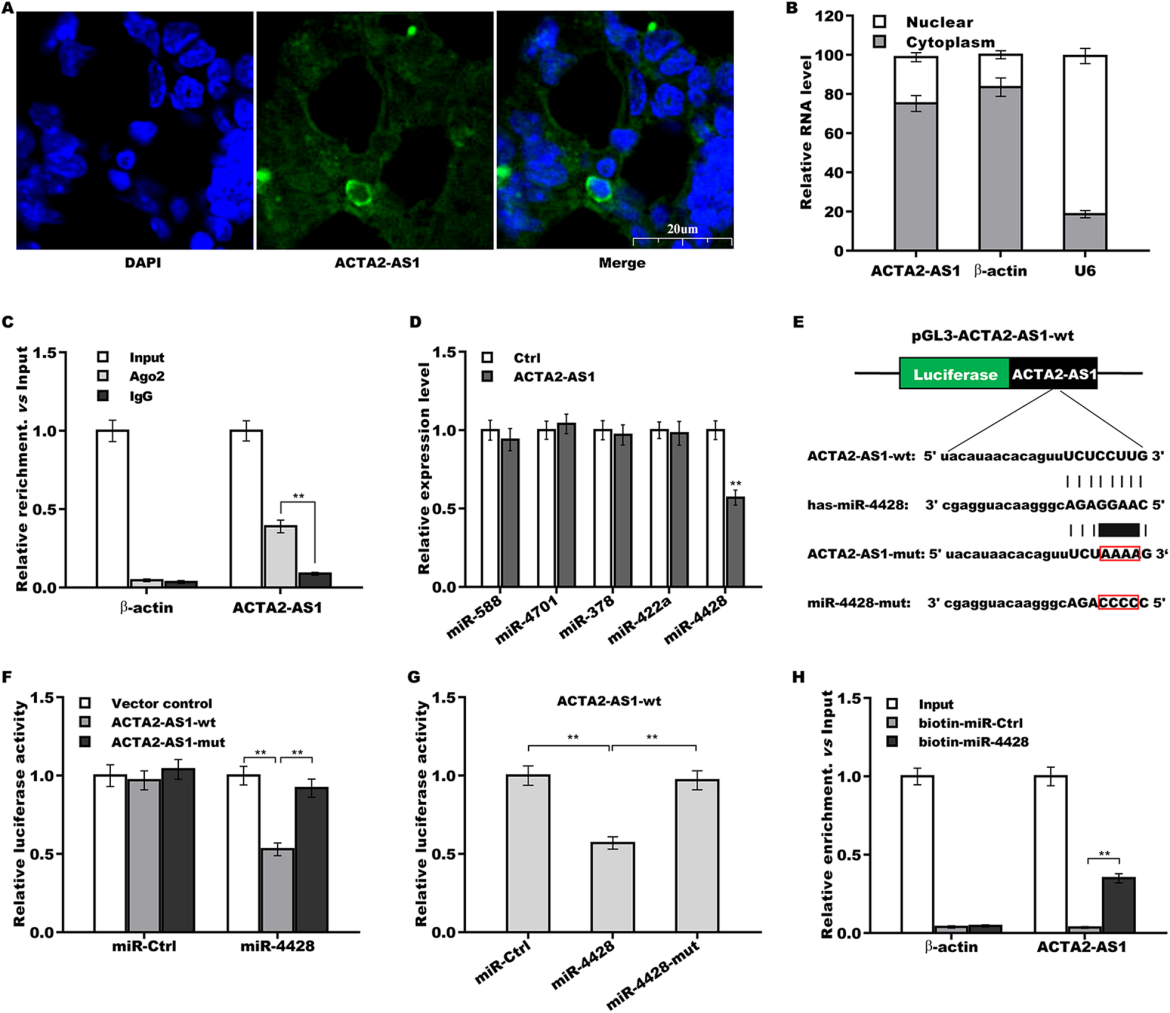
ACTA2-AS1 functioned as a ceRNA in sponging miR-4428. **A** FISH analysis of ACTA2-AS1 subcellular localization in TPC1 cells. ACTA2-AS1 probes are stained in green and nuclei are stained in blue. Scale bar, 20 µm and magnification, 400 × . **B** Total RNA was extracted from the nucleus and cytoplasm following which the ACTA2-AS1 level was assessed using qRT-PCR. **C** A RNA immunoprecipitation (RIP) assay was performed with an anti-AGO2 antibody to assess the enrichment of ACTA2-AS1 in AGO2-immunoprecipitated complexes in TPC1 cells. IgG served as a negative control; 10% of the total RNA was used as input. **D** MiRNA expression in ACTA2-AS1-overexpressing TPC1 cells was assessed by qRT-PCR. **E** Sequence analysis revealed that ACTA2-AS1 harbors a complementary miR-4428 target site. **F** pGL3-ACTA2-AS1-wt or pGL3-ACTA2-AS1-mut and miR-4428 were co-transfected into TPC1 cells and then luciferase activity was measured. **G** pGL3-ACTA2-AS1-wt and miR-4428 or miR-4428-mut were co-transfected into TPC1 cells and then luciferase activity was measured. **H** RNA pull-down was carried out with biotin-labeled miR-4428 to assess the enrichment of ACTA2-AS1 in miR-4428-immunoprecipitated complexes in TPC1 cells. **p < 0.01

LncRNAs located in the cytoplasm frequently act as ceRNAs, sponging miRNAs through miRNA response elements (MREs), thereby regulating mRNA expression [24, 25]. We next assessed cellular localization of ACTA2-AS1 using a FISH assay. As demonstrated in Fig. 4A, ACTA2-AS1 was found to predominantly localize to the cytoplasm in TPC1 cells, which was further confirmed via qRT-PCR (Fig. 4B). Furthermore, a RIP assay using an anti-AGO2 antibody showed that ACTA2-AS1 was highly enriched in AGO2-immunoprecipitated complexes (Fig. 4C), suggesting that ACTA2-AS1 might act as a ceRNA and, consequently, a miRNA sponge. To investigate this possibility, the starBase platform (http://starbase.sysu.edu.cn/) was employed to predict miRNAs which may be sponged by ACTA2-AS1, leading to the identification of five putative miRNAs—miR-588, miR-4701, miR-378, miR-422a, and miR-4428 (Additional file 2: Table S2). Subsequently, in TPC1 cells, ACTA2-AS1 overexpression was found to significantly decrease the expression of miR-4428, but not that of the other four miRNAs (Fig. 4D).

Given that ACTA2-AS1 harbors a complementary miR-4428 target site (Fig. 4E), we then assessed whether ACTA2-AS1 functions as a sponge of miR-4428 via the binding of miR-4428 seed region. For this, recombinant plasmids containing the complementary miR-4428 target site in ACTA2-AS1 (pGL3-ACTA2-AS1-wt and its mutant form pGL3-ACTA2-AS1-mut) were constructed. Each plasmid was separately co-transfected with miR-4428 into TPC1 cells, and then the effect on luciferase activity was assessed. The results showed that luciferase activity was markedly suppressed when miR-4428 was co-transfected with pGL3-ACTA2-AS1-wt; in contrast, luciferase activity was lost when miR-4428 was co-transfected with pGL3-ACTA2-AS1-mut, which harbors a four-nucleotide mutation in the complementary miR-4428 target site (Fig. 4F). Moreover, the mutation of four nucleotides in miR-4428 seed region also led to the complete suppression of pGL3-ACTA2-AS1-wt-related luciferase activity (Fig. 4G). Finally, the results of RNA pull-down with biotin-labeled miR-4428 showed that ACTA2-AS1 was highly enriched in miR-4428-immunoprecipitated complexes (Fig. 4H).

### Enhancement of MiR-4428 in the invasive capacity of PTC cells via regulating KLF9 expression

**Fig. 5:**
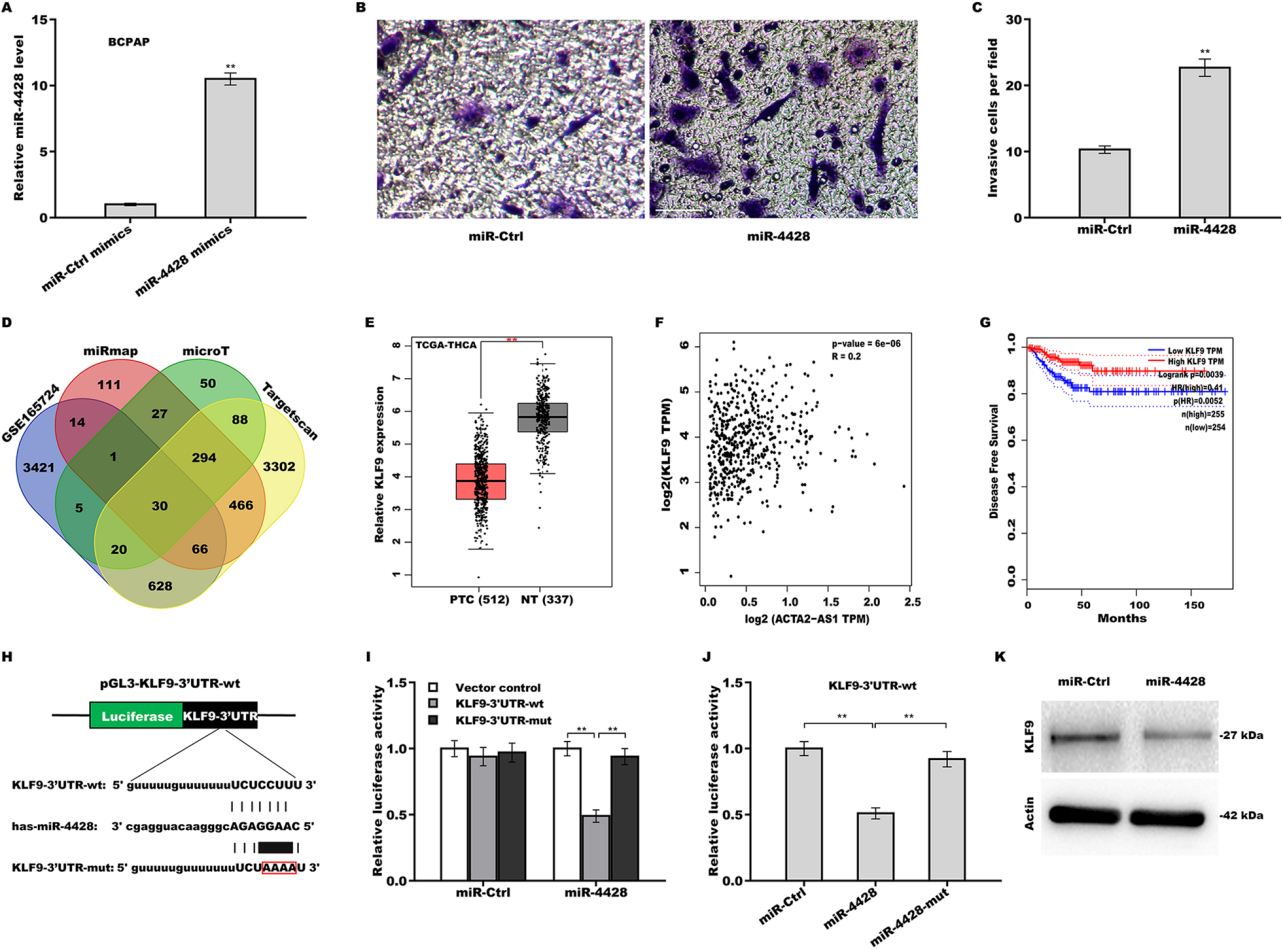
MiR-4428 enhanced the invasive capacity of PTC cells by regulating KLF9 expression. **A** The levels of miR-4428 in BCPAP cells treated with miR-4428 mimics were assessed by qRT-PCR. **B** Transwell invasion assay for the invasive ability of miR-4428-overexpressing BCPAP cells and **C** the respective quantitative analysis. Scale bar, 50 µm and magnification, 200× . **D** Three bioinformatics tools (TargetScan, miRmap, and microT, respectively) and the GSE165724 dataset were employed for the prediction of miR-4428 target genes. **E** The KLF9 level was analyzed in 512 PTC tissues and 337 normal tissues in The Cancer Genome Atlas (TCGA) and Genotype-Tissue Expression (GTEx) datasets from GEPIA. **F** The correlation between ACTA2-AS1 and KLF9 levels based on information from the TCGA and GTEx datasets from GEPIA. **G** Analysis of the correlation between KLF9 expression and disease-free survival using the GEPIA tool. **H** Sequence analysis revealed that *KLF9* harbors a complementary miR-4428 target site. **I** pGL3-KLF9-3’UTR-wt or pGL3-*KLF9-3’UTR-mut* was co-transfected with *miR-4428* into TPC1 cells following which luciferase activity was measured. **G** pGL3-KLF9-3’UTR-wt and miR-4428 or miR-4428-mut were co-transfected into TPC1 cells and then luciferase activity was measured. **K** The KLF9 protein level was measured by western bot in miR-4428-overexpressing BCPAP cells. **p < 0.01

As shown in Fig. 5A–C, we found that miR-4428 overexpression led to a significant increase in invasive potential of BCPAP cells. We then sought to identify miR-4428 target gene using both bioinformatic analysis and functional experiments. Three bioinformatics tools (TargetScan, microT, and miRmap) and the GSE165724 dataset were used to predict the miR-4428 target genes. Venn diagram analysis identified 30 genes as being miR-4428 targets in all four sets of data (Fig. 5D, Additional file 3: Table S3). Among the 30 genes, *KLF9* was selected for further study based on the following four reasons: (i) *KLF9* expression was lower in PTC tissues than in matched normal tissues in GSE165724 dataset [26] and *KLF9* is a known tumor suppressor; [27–29] (ii) *KLF9* expression was markedly lower in thyroid carcinoma tissues than in normal tissues in TCGA/GTEx datasets from GEPIA (Fig. 5E); (iii) the level of KLF9 was positively correlated with that of ACTA2-AS1 in TCGA/GTEx datasets (Fig. 5F); and (iv), low KLF9 expression was significantly correlated with worse disease-free survival in thyroid cancer patients (Fig. 5G).

*KLF9* harbors a complementary miR-4428 target site (Fig. 5H). To assess whether *KLF9* is a direct target of miR-4428, recombinant plasmids containing the complementary miR-4428 target site in the KLF9-3’UTR (pGL3-KLF9-3’UTR-wt and its mutant form pGL3-KLF9-3’UTR-mut) were then constructed. Each plasmid was separately co-transfected with miR-4428 into TPC1 cells and then the effect on luciferase activity was determined. As shown in Fig. 5I, the luciferase activity was greatly suppressed when miR-4428 was co-transfected with pGL3-KLF9-3’UTR-wt, whereas the opposite of luciferase activity was observed when miR-4428 was co-transfected with pGL3-KLF9-3’UTR-mut, which contains four mutated nucleotides in the complementary miR-4428 target site. Moreover, the mutation of four nucleotides in miR-4428 seed region also completely abrogated its suppressive effect (Fig. 5J). Importantly, miR-4428 overexpression markedly inhibited KLF9 protein expression in BCPAP cells (Fig. 5K).

### Role of ACTA2-AS1 in KLF9 overexpression and suppression of the invasive ability of PTC cells in a miR-4428-dependent manner

**Fig. 6:**
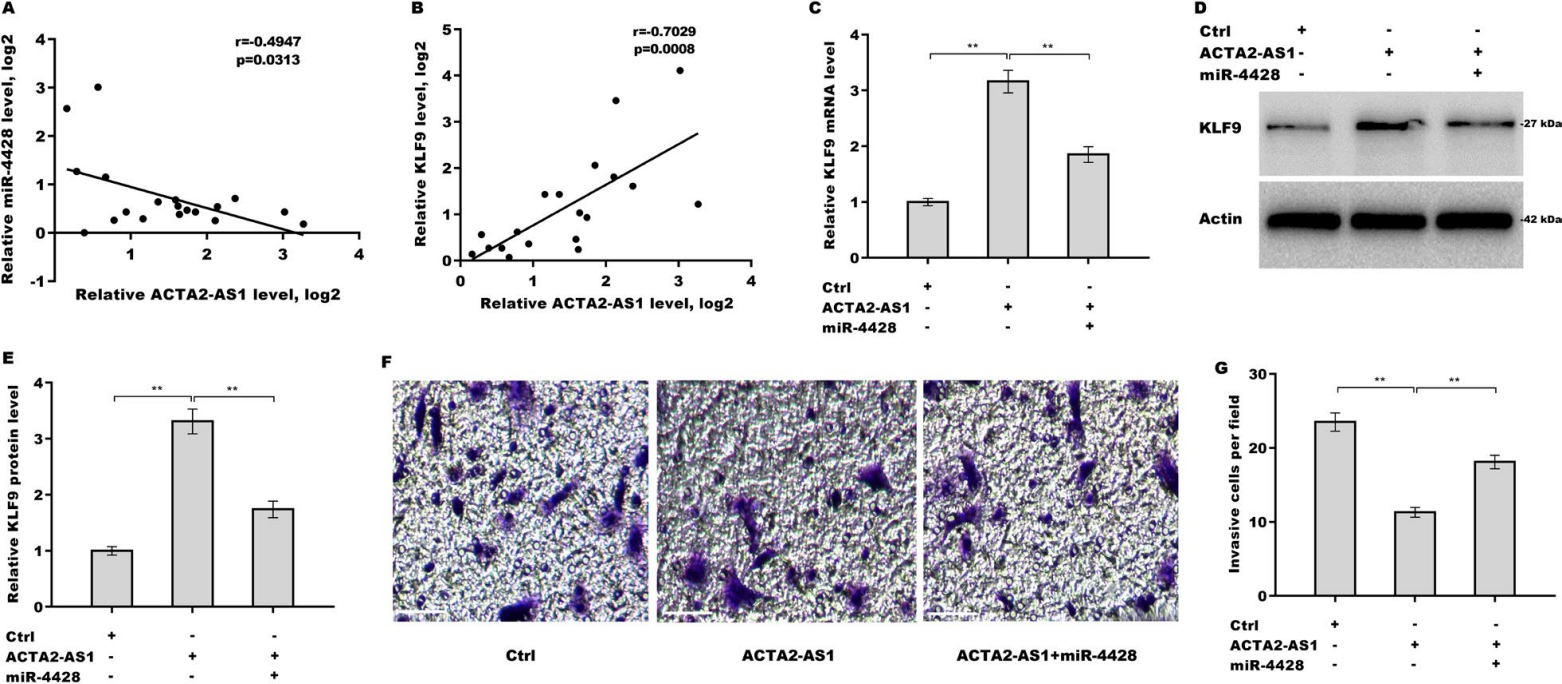
ACTA2-AS1 increased KLF9 expression and inhibited the invasive ability of PTC cells in a miR-4428-dependent manner. **A** qRT-PCR was employed to determine the KLF9 mRNA level in TPC1 cells overexpressing ACTA2-AS1 and miR-4428. **B**, **C**, **D**, **E** Western blot and quantitative analysis, respectively, of the KLF9 protein level in TPC1 cells overexpressing ACTA2-AS1 and miR-4428. **F**, **G** Transwell invasion assay and quantitative analysis, respectively, for the invasive ability of TPC1 cells overexpressing ACTA2-AS1 and miR-4428. Scale bar, 50 µm and magnification, 200× . **p < 0.01

Finally, we explored whether ACTA2-AS1 regulated KLF9 expression, thereby influencing the invasive capacity of PTCs, by sponging miR-4428. We found that ACTA2-AS1 overexpression increased KLF9 mRNA level, on which an effect was reversed by miR-4428 application (Fig. 6A). MiR-4428 also inhibited promotive effect of ACTA2-AS1 on KLF9 protein expression (Fig. 6B, C). Functionally, we found that ACTA2-AS1 overexpression reduced invasive ability of TPC1 cells, whereas miR-4428 significantly reversed this effect (Fig. 6D, E, F, G). Taken together, these results showed that ACTA2-AS1 inhibits PTC metastasis by regulating the miR-4428/KLF9 axis.

### Methylation-expression correlation

**Fig. 7:**
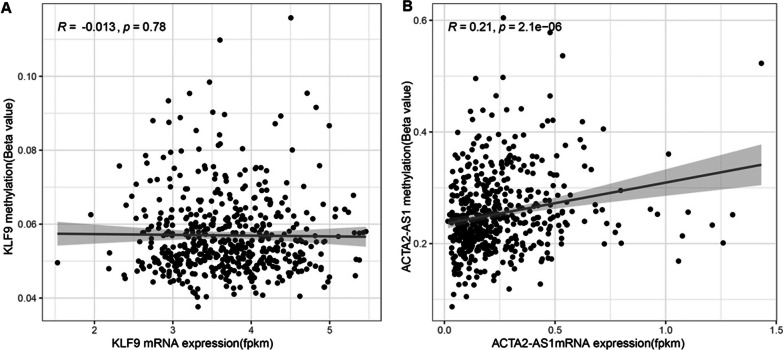
**A** Correlation between KLF9 expression and methylation in the TCGA dataset. **B** Correlation between ACTA2-AS1 Expression and methylation in the TCGA dataset

In the current study, we also further investigated the methylation data and mRNA expression data of 570 patients with PTC from the TCGA dataset. Our results indicated that no significant correlation between promoter methylation and mRNA expression of KLF9 gene was observed (*r* = -0.013, *p* = 0.78) (Fig. 7A), while the methylation level of ACTA2-AS1 was significantly correlated with its mRNA expression (*r* = 0.21, *p* = 2.1˟e^−6^) (Fig. 7B). Unfortunately, we could not show the similar correlation results for miR-4428 as the methylation data on this gene are not available in the TCGA dataset.

## Discussion

Increasing evidence has indicated that lncRNAs are frequently dysregulated in most types of cancers and play important roles in tumor progression [30]. Bian et al. demonstrated that 496 lncRNAs were differentially expressed between colorectal cancer tissues and normal tissues. They further reported that the elevated lncRNA-FEZF1-AS1 expression accelerated colorectal cancer growth and metastasis through overexpression of pyruvate kinase 2 [31]. Similarly, Feng et al. found that 568 lncRNAs were aberrantly expressed in PTC tissues and that the increased levels of lncRNA-n384546 facilitated PTC growth and metastasis via regulation of the miR-145/AKT3 axis [32]. Recent studies have shown that ACTA2-AS1 exerts a tumor suppressive effect in a variety of cancers [16–19]. However, whether ACTA2-AS1 plays a role in PTC metastasis remains unknown.

In the current study, we demonstrated that ACTA2-AS1 expression is decreased in PTC and that ACTA2-AS1 inhibits proliferative and invasive abilities of PTC cells. We further demonstrated that ACTA2-AS1 acts as a ceRNA by sponging miR-4428. Meanwhile, ACTA2-AS1 expression was found to increase KLF9 expression and inhibit the invasive ability of PTC cells in a miR-4428-dependent manner. These results reveal the role of the ACTA2-AS1/miR-4428/KLF9 axis in PTC metastasis and represent a promising opportunity for the development of PTC-targeting therapy.

CeRNAs comprise a class of cytoplasmic decoys that compete with a common pool of miRNAs. Through MREs, ceRNAs can regulate the expression of genes via a complex network that includes non-coding RNAs (lncRNAs and miRNAs) and mRNAs [23]. It was thought that all MRE-containing lncRNAs can sequester miRNAs and seclude them from mRNAs containing the same MREs, thereby increasing mRNA transcript levels [23, 33]. Studies have revealed that lncRNAs exert their tumorigenic effects on human tumors by acting as ceRNAs. For instance, LINC01133 inhibits epithelial–mesenchymal transition in gastric cancer cells and cancer metastasis via functioning as a sponge of miR-106a, thereby increasing the levels of adenomatous polyposis coli (APC) [34]. Similarly, ACTA2-AS1 can inhibit the growth of colon cancer cells via sponging miR-4428 and thus promote BIM expression [16]. Besides miR-4428, ACTA2-AS1 can also function as a sponge of other miRNAs, such as miR-532 and miR-143 [35, 36]. Additionally, ACTA2-AS1 can regulate gene expression through other epigenetic mechanisms. Navarro-Corcuera et al. demonstrated that ACTA2-AS1 can increase platelet-derived growth factor B (PDGFB) expression by recruiting p300/ELK1 to the PDGFB promoter, thereby triggering the acetylation of lysine 27 on histone H3 (H3K27ac) [37].

In this study, we investigated the roles of ACTA2-AS1 in PTC metastasis and sought to identify the underlying mechanisms. We found that ACTA2-AS1 expression was continuously downregulated in normal tissues, PTC tissues without metastasis, and PTC tissues with lymph node metastasis, respectively. Functionally, we found that overexpression of ACTA2-AS1 decreased the growth of TPC1 cells and their proliferative as well as invasive capacities. To identify the mechanism by which ACTA2-AS1 inhibits PTC metastasis, we further assessed subcellular localization of ACTA2-AS1 in PTC cells, showing that ACTA2-AS1 majorly localized in the cytoplasm. We further found that ACTA2-AS1 was highly enriched in AGO2-immunoprecipitated complexes, indicating that ACTA2-AS1 might function as a ceRNA. The bioinformatic analysis and functional experiments demonstrated that ACTA2-AS1 suppressed PTC metastasis by sponging miR-4428, consequently, increasing KLF9 expression. Finally, Although BCPAP is derived from poorly differentiated thyroid carcinoma, many studies have used this cell line to investigate the migration and invasion of thyroid carcinoma in vitro [38–41]*.* For example, Cazarin, et al. showed that 5'-AMP-activated protein kinase regulates TPC-1 and BCPAP cell survival, migration, invasion, and epithelial-to-mesenchymal transition [38]. Awwad, et al. demonstrated that the AMPK-activator (AICAR) reduced the basal migration of TPC-1 and BCPAP but the similar finding did not observed in the normal human thyroid cell line (Nthy-ori 3–1) [39]. Chong et al. showed that knockdown of IL13RA2 in BCPAP cells significantly reduced cell viability, cell migration, and epithelial-to-mesenchymal transition [40]. Furthermore, Xu, et al. demonstrated that the activation of TRPV1 by capsaicin significantly suppressed the migration and invasion of BCPAP cells as well as their adhesion [41]. Taken together, such evidence may demonstrate that BCPAP cells could be used as a cell model in thyroid carcinoma metastasis.

Previous studies have suggested that epigenetic modification (e.g., DNA methylation) of activating proto-oncogenes and inhibiting tumor suppressor genes is one of the most important events in malignancies, including PTC [42, 43]. Abnormal DNA methylation and expression levels of ACTA2-AS1 were demonstrated in several tumors [44]. Using the TCGA dataset, we observed that the ACTA2-AS1 promoter methylation level was positively correlated with its mRNA expression. Thus, this finding suggests that DNA methylation of ACTA2-AS1 could be one of mechanisms in the regulation of ACTA2-AS1 expression in PTC.

**Fig. 8:**
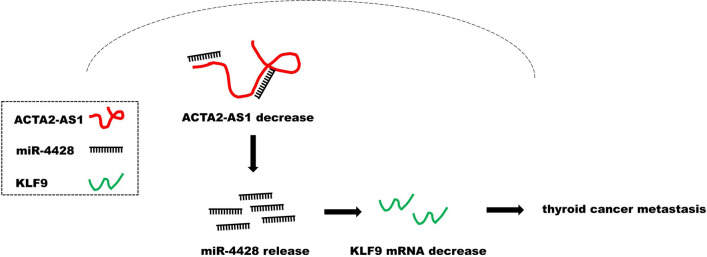
Graphical abstract

In conclusion, our findings indicate that ACTA2-AS1 may function as a tumor suppressor in PTC, at least partly by regulating miR-4428-dependent KLF9 expression as demonstrated in the graphical abstract (Fig. 8).

## Materials and methods

### Clinical specimens

Nineteen fresh PTC specimens without metastasis and matched normal thyroid tissues, as well as 11 PTC specimens with lymph node metastasis, were collected with the approval of the Shanghai Baoshan Hospital of Integrated Traditional Chinese and Western Medicine Ethics Committee (No. 201917). The written informed consent was obtained from all the donors. The clinical characteristics of the patients are shown in Table 1. None of the donors had received therapy before surgery.

**Table 1:** Correlation of ACTA2-AS1 level with clinicopathologic features in PTC

Variable (*n* = 30)		ACTA2-AS1 level		*p* value
		High	Low	
Age (years)	≤ 55	8	10	0.765
	> 55	6	6	
Gender	Female	8	9	0.961
	Male	6	7	
Tumor size (cm)	≤ 2	7	7	0.732
	> 2	7	9	
PTC subtype	Classic	9	9	0.654
	Encapsulated classic	5	7	
TNM stage	I + II	12	6	0.007*
	III + IV	2	10	
LN metastasis	No	12	7	0.017*
	Yes	2	9	

*PTC* papillary thyroid carcinoma; *LN*, lymph node

*Statistically significant (*χ*2 test)

### Cell culture

The human PTC cell lines BCPAP and TPC1 and the normal human thyroid cell line Nthy-ori 3–1 (hereafter abbreviated as Nthy) (Procell Life Science & Technology Co, Ltd, Wuhan, China) were maintained in RPMI 1640 medium (Solarbio, Beijing, China) supplemented with 10% fetal bovine serum (Cellmax, Beijing, China), 100 U/mL penicillin, and 0.1 mg/mL streptomycin (Solarbio) at 37 °C with 5% CO2.

### Cell transfection

Recombinant constructs containing ACTA2-AS1 cDNA (pcDNA-ACTA2-AS1) were established by cloning the *ACTA2-AS1* gene into the pcDNA3.1 vector. Small interfering RNAs (siRNAs) targeting *ACTA2-AS1* (si-ACTA2-AS1s, a mixture of three siRNAs) and the negative control were designed and synthesized by Biosynthech (Jiangsu, China). MiR-4428 mimics and control miRNA (miR-Ctrl) mimics were purchased from Sangon (Shanghai, China). TPC1 and BCPAP cells in the logarithmic growth phase were grown in a culture dish until 60–80% confluency, following which the indicated plasmids, siRNAs, and miRNA mimics were transfected into the cells using Lipofectamine 3000 (Invitrogen, CA, USA) according to the manufacturer's instructions. The sequences of the miRNAs and siRNAs used in this study are shown in Additional file 1: Table S1.

### qRT-PCR

Total RNA was extracted from PTC cells or fresh PTC tissues with TRIzol reagent (Sigma-Aldrich, MO, USA) and quantified in a Varioskan LUX microplate reader (Thermo Fisher Scientific, MA, USA). Cytoplasmic and nuclear RNA was extracted from thyroid carcinoma cells using the Cytoplasmic & Nuclear RNA Purification Kit from AmyJet Scientific (Wuhan, China). Reverse transcription was performed using the TIANScript II RT Kit (Tiangen, Beijing, China) at 42 °C. The qPCR was performed using StarLighter SYBR Green qPCR Mix (Qihengxing, Beijing, China) in the QuantStudio 5 Food Safety Real-Time PCR System (Thermo Fisher Scientific). The thermocycling conditions were set at 95 °C for 5 min followed by 37 cycles of 95 °C for 20 s and 58.5 °C for 20 s. The sequences of all the primers (Genewiz, Jiangsu, China) used in this study are shown in Additional file 1: Table S1. The relative levels of ACTA2-AS1 were calculated using β-actin as the internal reference gene. The *U6* served as the internal reference gene for the determination of miRNA levels.

### Colony formation assay

After treatment with the indicated reagents, a total of 400 cells were seeded into six-well plates. After 14 days, the colonies were rinsed with PBS, fixed in 4% paraformaldehyde (PFA), and stained with 0.1% crystal violet for 18 min. Colonies > 50 μm in diameter were counted using ImageJ (NIH, ND, USA).

### Cell proliferation assay

A cell counting Kit-8 (CCK-8) assay was applied to assess the proliferative potential of cells after ACTA2-AS1 overexpression or knockdown. Approximately 5,000 TPC1 or BCPAP cells were seeded in 96-well plates and allowed to attach at 37 °C overnight. The cells were then transfected with the indicated plasmids or siRNAs and cultured for the indicated times, after which CCK-8 reagent (10 µL) was applied to the cells for 60 min. Finally, the optical density (OD) was measured at a wavelength of 450 nm using a microplate reader.

### Transwell invasion assay

BCPAP and TPC1 cells were treated with pcDNA-ACTA2-AS1, si-ACTA2-AS1s, or miR-4428 mimics and the invasive potential of the cells was assessed using 8-μm-pore Transwell inserts (X-bio Technology, Nanjing, China). The upper chambers of the inserts were coated with Matrigel (Corning, NY, USA) for 12 h at 37 °C. Cells were placed in the upper chamber in serum-free medium while RPMI 1640 medium containing 10% FBS was added to the lower chamber. After 24 h of incubation, the cells on upper surface of the membrane (unmigrated cells) were wiped off, and the upper chamber was washed twice with PBS. The cells that crossed the membrane (migrated cells) were fixed in 4% PFA, stained with 0.1% crystal violet, and counted under a microscope.

### Fluorescence in situ hybridization (FISH)

The subcellular localization of ACTA2-AS1 in TPC1 cells was assessed using the FISH Tag RNA Multicolor Kit (Thermo Fisher Scientific). TPC1 cells were plated onto 14-mm-diameter glass coverslips, washed with PBS, fixed in 4% PFA, and incubated with proteinase K (2 μg/mL), glycine, and acetic anhydride at 37 °C. After pre-hybridization for 60 min, the cells were hybridized with probe targeting ACTA2-AS1 at 37 °C, counterstained with DAPI for 5 min, and finally imaged with a XSP-63B fluorescence microscope (Shanghai Optical Factory No. 1, Shanghai, China).

### In vivo metastasis assay

All the procedures with animals were performed with the approval of the Experimental Animal Committee of Shanghai University of Traditional Chinese Medicine (PZSHUTCM210820017). Male athymic Balb/c mice (6–8 weeks old) were obtained from Cyagen Biosciences (Jiangsu, China) and housed in a specific pathogen free room (12-h-light/12-h-dark cycle, 22 ± 2 °C) with ad libitum access to standard chow. To establish the metastasis model, TPC1 cells stably expressing ACTA2-AS1 (3 × 10^6^/200 μL PBS) were injected into the mice via the tail vein. Mice showing signs of unbearable pain or disease were euthanatized before the end of the experiment. In vivo bioluminescent imaging of lung metastasis was performed at the 9th week after injection. All the mice were subsequently euthanized via inhalation anesthesia (2% isoflurane) followed by cervical dislocation. Lung tissues were dissected, fixed in 4% PFA, and stained with hematoxylin and eosin (H&E). Finally, the numbers of metastatic nodules were counted.

### RNA immunoprecipitation (RIP) assay

The RIP assay was carried out using a commercial RIP kit (BersinBio, Guangzhou, China) and antibodies against IgG (SPA201; Solarbio) and AGO2 (FNab10000; FineTest, Hubei, China). TPC1 cells were lysed with NP-40 Lysis Buffer (Solarbio) and centrifuged at 13,000 × *g* for 5 min. The collected supernatant was incubated with 300 μL of RIP buffer containing beads coated with the indicated antibodies for 12 h at 4 °C. After centrifugation at 3,000 × *g* for 60 s, the supernatant was discarded and the beads were washed five times with PBS, followed by digestion with proteinase K. RNA was extracted using TRIzol reagent and subjected to qRT-PCR.

### RNA pull-down

Biotin-labeled miR-4428 (GenePharma, Shanghai, China) was incubated with streptavidin agarose beads at room temperature (RT) for 60 min. A TPC1 cell lysate was mixed with probe-coated beads at 4 °C for 12 h. RNA that was bound by miR-4428 was purified using TRIzol and quantified via qRT-PCR.

### Dual-luciferase reporter assay

The pGL3-ACTA2-AS1-wt and pGL3-KLF9-3’-UTR-wt recombinant plasmids or their mutant forms (pGL3-ACTA2-AS1-mut and pGL3-KLF9-3’-UTR-mut, respectively) were generated by inserting predicted or mutated binding sites of KLF9-3’UTR or ACTA2-AS1 into the pGL3 vector (VectorBuilder, Guangzhou, China). A total of 1 × 10^5^ TPC1 cells were seeded into a 24-well plate and co-transfected with 50 nM of miR-4428 or miR-Ctrl; 20 ng of pGL3-ACTA2-AS1-wt, pGL3-KLF9-3’-UTR-wt, or either of their mutant forms; and 2 ng of pRL-TK (VectorBuilder) using Hieff Trans Liposomal Transfection Reagent (Yeasen, Shanghai, China). For two days after transfection, the cells were lysed and monitored for luciferase activity using the Dual-Luciferase Reporter Gene Assay Kit (Yeasen).

### Western blot

The cells were homogenized in NP-40 Lysis Buffer (Solarbio) containing a protease inhibitor cocktail. After determination of the protein concentration using a BCA protein assay kit (Thermo Fisher Scientific), approximately 30 μg of protein from each sample was separated on a Future PAGE 10% gel (ACE Biotechnology, Jiangsu, China) and transferred onto PVDF membranes. The membranes were then blocked with 2% BSA and incubated with anti-KLF9 antibody (1:1,000; ab227920; Abcam, CA, USA) and anti-β-actin antibody (1:3,000; SAB5600204; Sigma-Aldrich) at 4 °C for 18 h. After rinsing three times with TBST, the membranes were incubated with horseradish peroxidase (HRP)-conjugated anti-rabbit IgG antibody (1:5,000; #7074S; Cell Signaling, MA, USA) at RT for 90 min. The immunoblots were visualized using Super ECL Detection Reagent (Yeasen).

### Data validation using the TCGA dataset

We analyzed DNA methylation and mRNA expression in the Cancer Genome Atlas (TCGA) database by the R version 4.21 software [Package: ggplot2]. The obtained data were retrieved and analyzed in UCSC Xena Functional Genomics Explorer (https://xenabrowser.net/).

### Statistical analysis

All data were obtained in experiments revealed from > 3 individual repeats and are presented as means ± standard error of mean (SEM). We quantified and classified the ACTA2-AS1 into low or high expression using a 50% cut-off level. Paired Student’s *t*-tests and ANOVA were used for statistical analysis. The Pearson correlation was used to explore the correlation between DNA methylation and mRNA expressions of these genes. The *p* value < 0.05 was considered to be significant.

### Supplementary Information

**Additional file 1**: **Table S1**. Primer, siRNA, and miRNA sequence used in the study**.**

**Additional file 2**: **Table S2**. Targeted miRNAs of ACTA2-AS1 predicted by the Starbase platform**.**

**Additional file 3**: **Table S3**. Target genes of miR-428 predicted by GSE165724, microT, miRmap, and Targetscan tool, respectively**.**

## Data Availability

The datasets and materials used and/or analyzed during the current study are available from the corresponding author on reasonable request.

## Abbreviations

TC: Thyroid carcinoma
lncRNAs: Long non-coding RNAs
PTC: Papillary thyroid cancer
miRNAs: MicroRNAs
ceRNAs: Competing endogenous RNAs
siRNAs: Small interfering RNAs
miR-Ctrl: Control miRNA
CCK-8: Cell Counting Kit-8
OD: Optical density
PFA: Paraformaldehyde
H&E: Hematoxylin and eosin
RIP: RNA immunoprecipitation
SEM: Standard error of mean
TCGA: The Cancer Genome Atlas
GTEx: Genotype-Tissue Expression
MREs: MiRNA response elements
FISH: Fluorescence in situ hybridization
APC: Adenomatous polyposis coli
