# Supplementary material for: Long non-coding RNA ACTA2-AS1 suppresses metastasis of papillary thyroid cancer via regulation of miR-4428/KLF9 axis
Source: Clin Epigenetics. 2024 Jan 9;16:10. doi: 10.1186/s13148-023-01622-6 (PMC10775490; doi:10.1186/s13148-023-01622-6)
Supplement: Supplementary file 1 — Additional file 1: Table S1. Primer, siRNA, and miRNA sequence used in the study. [file 13148_2023_1622_MOESM1_ESM.docx]

### **Supplementary Table S1. Primer, siRNA, and miRNA sequence used in the study.**

| **primer** | **Sense (5’-3’)** | **Antisense (5’-3’)** |
| --- | --- | --- |
| For  pcDNA-ACTA2-AS1 | ccaagcttggGTGCTTAGGCACTGCAGTTGAG | ggaatcccCCTTTCTCTAGCTAGCTCATTT |
| siRNA for  ACTA2-AS1-1# | GGAGAAACUGUGUUAUGUAGC | UACAUAACACAGUUUCUCCUU |
| siRNA for  ACTA2-AS1-2# | CAUGAAGAUCAAGAUCAUUGC | AAUGAUCUUGAUCUUCAUGGU |
| siRNA for  ACTA2-AS1-3# | CCAUGAAGAUCAAGAUCAUUG | AUGAUCUUGAUCUUCAUGGUG |
| Has-miR-4428 mimic | CAAGGAGACGGGAACAUGGAGC | |
| qRT-PCR for  ACTA2-AS1 | AGAGACAGAGAGGAGCAGGAAAG | ATGTGGATCAGCAAACAGGAATA |
| RT-PCR for  miR-4428 | GTCGTATCCAGTGCAGGGTCCGAGGTATTCGCACTGGATACGACgctcc | |
| qRT-PCR for  miR-4428 | CGCGCAAGGAGACGGGAACAT | GTGCAGGGTCCGAGGT |
| qRT-PCR for  KLF9 | TACCGACCCATCCAGACCCCCT | TGTTTCCCCTTCGCAGCCACTC |
